# The rising incidence of *Acanthamoeba keratitis*: A 7-year nationwide survey and clinical assessment of risk factors and functional outcomes

**DOI:** 10.1371/journal.pone.0222092

**Published:** 2019-09-06

**Authors:** Anna C. Randag, Jeroen van Rooij, Arnoud T. van Goor, Samuël Verkerk, Robert P. L. Wisse, Isabelle E. Y. Saelens, Remco Stoutenbeek, Bart T. H. van Dooren, Yanny Y. Y. Cheng, Cathrien A. Eggink

**Affiliations:** 1 Rotterdam Eye Hospital, Rotterdam, the Netherlands; 2 Radboud University Medical Center, Nijmegen, the Netherlands; 3 University Medical Center Utrecht, Utrecht, the Netherlands; 4 University Eye Clinic, Maastricht University Medical Center, Maastricht, the Netherlands; 5 University Medical Center Groningen, Groningen, the Netherlands; 6 Amphia Hospital, Breda, the Netherlands; 7 Erasmus Medical Center Rotterdam, Rotterdam, the Netherlands; 8 Leiden University Medical Center, Leiden, the Netherlands; University of California, UNITED STATES

## Abstract

**Purpose:**

To evaluate the incidence of *Acanthamoeba* keratitis in the Netherlands between 2009 and 2015 and to analyse predicting factors for treatment outcome.

**Methods:**

Patient characteristics, diagnostic methods, diagnostic delay, therapy prior to and after diagnosis, and visual outcome were obtained from medical files of all patients diagnosed with *Acanthamoeba* keratitis in the Netherlands between 2009 and 2015. A logistic regression analysis on treatment failure, defined as a best corrected visual acuity of less than 20/40 Snellen decimals (i.e. >0.3 logMAR or an approximate loss of three lines of visual acuity) and/or the need for keratoplasty, was performed to determine predicting factors.

**Results:**

Two hundred and twenty-four eyes of 224 patients were included. Ninety-five percent of the patients were contact lens wearers, of whom 74% wore soft contact lenses. The number of cases increased from 16 in 2009 to 49 in 2015. This resulted in an estimated incidence of 1 in 21,000 for soft contact lens wearers in 2015. Eighty-seven eyes (39%) met the criteria for treatment failure. In a multivariable regression analysis, higher age at presentation, a higher severity stage and corticosteroid use before diagnosis were positively correlated with treatment failure. Early referral to a cornea specialist was associated with better clinical outcomes.

**Conclusions:**

Although *Acanthamoeba* keratitis is still a relatively uncommon disease, the incidence in soft contact lens wearers has increased to reach 1 in 21,000 in 2015. Treatment failure occurred in 39% of cases, with age, higher severity stage, corticosteroid use before diagnosis and indirect referral to a cornea specialist as important risks factors.

## Introduction

*Acanthamoeba* keratitis (AK) is a rare, but potentially blinding infection of the cornea. *Acanthamoeba* species are ubiquitous, free-living amoebae, commonly found in soil and water, including tap water. *Acanthamoeba* infections can lead to the loss of an eye when untreated, but even after treatment, AK can cause significant loss of vision.

AK was first described in 1974 and its incidence rose during the following years, concurrently with the increase of contact lens wear, which was identified as a major risk factor [1]. In 2003, Seal estimated an annual incidence rate of one case of AK per 30,000 soft contact lens wearers for Europe and Hong Kong, based on several cohort studies and surveys [2]. One of these studies was the last national survey of microbial keratitis in contact lens wearers in the Netherlands, performed prospectively during three months in 1996, in which only one case of AK was encountered [3]. Recently, Carnt et al. described an outbreak of AK in the south east of the United Kingdom, starting in 2010–2011 [4]. Recent incidence data of our country are lacking, but cornea specialists report a rise in incidence over the last decade as well. The primary goal of this study was to investigate whether the AK incidence is indeed increasing.

The diagnosis and treatment of AK is fraught with difficulties. A significant delay in AK diagnosis is not uncommon, because of its rarity and its initial clinical similarity to other infections, like herpes simplex keratitis. There is no international agreement on a standard therapy for AK, and likewise, national guidelines are lacking, although all cornea specialists agree on the use of chlorhexidine with or without an additional drop containing polyhexamethylene biguanide (PHMB) or propamidine isethionate (Brolene^®^). However, the response to AK therapy can be disappointing, because of the defence mechanism of Acanthamoeba to extreme conditions and therapeutical agents: once the amoeba is threatened, impermeable cysts are formed. The prognosis is therefore guarded. Two large national surveys in the United Kingdom show that surgical intervention by means of keratoplasty or enucleation was required in 8.5% and 13.2% of patients and a suboptimal visual outcome was not uncommon [5,6]. Besides updating our national incidence rates, the secondary purpose of this study was to find out which factors significantly influence treatment outcome in AK cases.

## Materials and methods

For this retrospective case series, data from all patients diagnosed with AK between 1 January 2009 and 31 December 2015 were collected, by means of an inquiry among all cornea specialists in the Netherlands in 2014 and 2016. The study adhered to the tenets of the Declaration of Helsinki and local laws on research with human subjects. Ethical approval was not required, as the data were collected retrospectively.

Cases were identified through medical microbiology results, pathology results and hospital diagnostic and administrative codes. The inquired information consisted of demographic patient characteristics, the course of keratitis before diagnosis (including corticosteroids use), clinical presentation at the time of diagnosis, method of diagnosis, medical treatment after diagnosis, surgical intervention, pain management and follow up visits. In order to minimise the effect of patient related factors, in cases with bilateral disease either the secondly affected eye, or a randomly chosen eye if presentation was simultaneously, was excluded from further analyses.

### Main outcome measures

Incidence numbers were based on the number of cases per year, compared to the total number of contact lens wearers in the Netherlands. The dependent variable for the regression analysis was ‘failure’ of primary AK treatment, defined as a last measured best corrected visual acuity (BCVA) of less than 20/40 Snellen decimals (i.e. >0.3 logMAR or an approximate loss of three lines of visual acuity) and/or the need for keratoplasty.

### Exposure factors

To classify the severity of AK at the time of diagnosis the classification described by Robaei et al. was used [7]. Stage 1 included only corneal epitheliopathy. For stage 2, at least one corneal epithelial defect, a perineural or a stromal infiltrate had to be present on top of stage 1. Stage 3 included findings of stage 2 with addition of a corneal ring infiltrate.

In absence of a numeric pain score, pain intensity was based on the maximally needed pain medication, assuming that the use of no medication correlated with a low pain intensity and the use of opioids matched with high pain intensity.

### Statistical analyses

A one-sample chi-square test was used to analyse seasonal variation. In order to find predictors for the outcome ‘treatment failure’, univariable as well as multivariable logistic regression models were built with ‘treatment failure’ as dependent variable. Variables that showed a relation in the univariable analysis (P < 0.1) were used in the multivariable models. Backward, forward and stepwise procedures were used to restrict the number of predictors to the most essential. All possible two-way interactions between the predictors were checked. Only two-sided tests were executed, in which a P-value smaller than 0.05 was considered to be statistically significant.

## Results

All fourteen specialized centres in the Netherlands, of which eight were academic hospitals, participated. Between 2009 and 2015, 224 patients with AK were identified in the Netherlands. Patient characteristics are presented in Table 1. Ten eyes from bilaterally affected patients (4.5%) were excluded, resulting in 224 eyes for further analyses. Nearly all patients (95%) were contact lens users, of whom 75% wore soft contact lenses. Herpes simplex virus (HSV) infection was suspected in 58% of cases, but only two cases turned out to have a laboratory confirmed HSV coinfection. Sixty-four percent of the eyes were treated with corticosteroids before diagnosis, with a median duration of 19 days. Delay until diagnosis was partly caused by patients (median 8 days between start of complaints and presentation to the clinic), but mostly by doctors (median 18 days between presentation and diagnosis). There were strong associations between doctor’s delay, HSV suspicion and use of corticosteroids before diagnosis (data not shown). AK diagnosis was confirmed by PCR, culture, confocal microscopy or pathology (either from biopsy or keratoplasty) in all but 17 cases (7.6%), in which the clinical characteristics and course after treatment were typical. A coinfection was found in 19%, predominantly with bacterial pathogens (Table 1).

**Table 1:** Patient characteristics (n = 224).

Characteristics	No. (%)
**Sex**	
Male	84 (37.5)
Female	140 (62.5)
**Age at presentation, years**	
Median	34
Range	11–75
Unknown, n	5
**Affected eye**	
Right	110 (49.1)
Left	102 (45.5)
Both	10 (4.5)
Unknown	2 (0.9)
**Contact lens wear**	
Yes	213 (95.1)
- Soft	158 (74.2)
- Rigid-gas permeable	41 (19.2)
- Other (including non-specified)	14 (6.6)
No	6 (2.7)
Unknown	5 (2.2)
**Referral by**	
Patient	8 (3.6)
General practitioner	50 (22.3)
Other ophthalmologist	161 (71.9)
Other	2 (0.9)
Unknown	3 (1.3)
**Patient’s delay, days**	
Median	8
Range	0–150
Unknown, n	27
**Doctor’s delay, days**	
Median	18
Range	0–288
Unknown, n	6
**Total delay, days**	
Median	29
Range	2–319
Unknown, n	27
**Herpes virus infection suspected**	
Yes	130 (58.0)
No	92 (41.1)
Unknown	2 (0.9)
**Corticosteroid use before diagnosis**	
Yes	143 (63.8)
No	77 (34.4)
Unknown	4 (1.8)
**Disease stage at diagnosis**	
Stage 1	58 (25.9)
Stage 2	95 (42.4)
Stage 3	64 (28.6)
Unknown	7 (3.1)
**Coinfection at diagnosis**	
None	182 (81.2)
Bacterial	26 (11.6)
Fungal	8 (3.6)
Bacterial and fungal	4 (1.8)
Viral	2 (0.9)
Unknown	2 (0.9)

### Incidence rates

**Fig 1:**
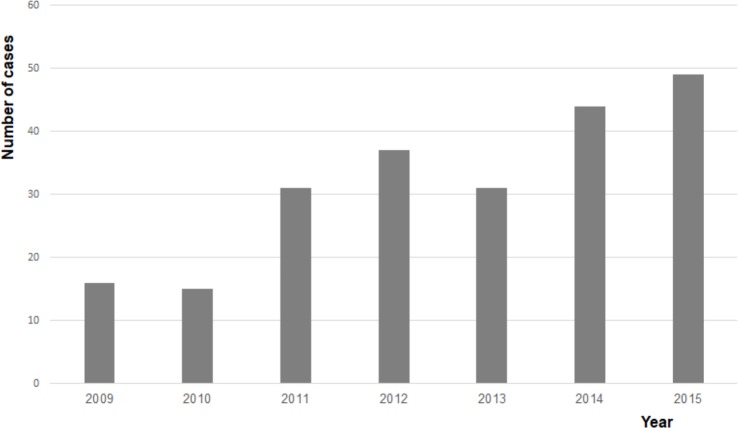
Number of cases per year. The annual number of cases of *Acanthamoeba* keratitis in the Netherlands.

**Fig 2:**
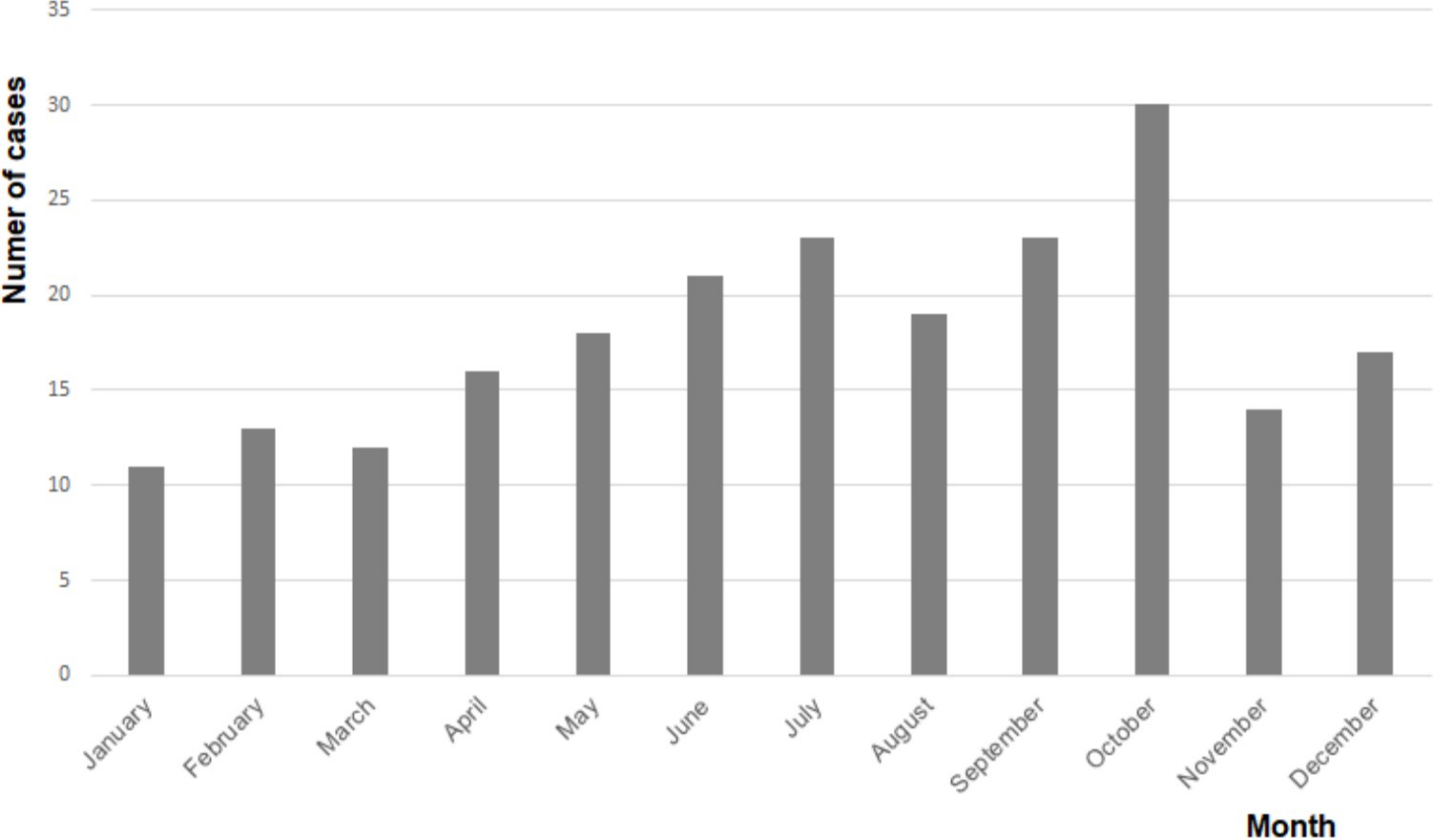
Number of cases per month. The cumulative number of cases of *Acanthamoeba* keratitis per month in the Netherlands, between 2009 and 2015.

The number of cases increased from 16 in 2009 to 49 in 2015, resulting in an average annual increase of 20.5% (Fig 1). Due to the small numbers, incidence rates could not be reliably calculated for 2009. In 2015, the total population of the Netherlands counted 16,900,726, of whom 11,065,975 were between 15 and 65 years of age [8]. According to data from Euromcontact, which are only available for this age group, 7.86% were soft contact lens wearers, resulting in an estimated incidence of 1 AK case in 21,000 soft contact lens wearers in 2015 [9]. Reliable data concerning the nationwide use of rigid gas permeable contact lenses were not available. Fig 2 shows that most cases occurred in summer and autumn, which was statistically confirmed (p = 0.028).

### Treatment and outcome

Table 2 outlines the treatment regimens started by the ophthalmologists. Most patients were treated with a combination therapy of chlorhexidine and Brolene^®^, however, monotherapy (mostly with chlorhexidine) was applied in 16%. In 50 patients (22.4%) an acute surgical intervention was required, because of keratitis not responding to treatment, progression of keratitis towards the limbus, or impending perforation: a therapeutic keratoplasty was performed in 49 cases and one eye was enucleated. Additionally, 34 operations were performed in a quiet phase of the keratitis: 32 keratoplasties (for visual rehabilitation) and 2 eviscerations. Cataract surgery was performed in at least 32 cases, with 26 cases of the whole population missing lens status data at final follow up. Eventually, 66 patients (29.5%) underwent one or more surgical interventions. After a median follow-up duration of 353 days (range 12–1682), a final BCVA could be retrieved for 208 eyes (93%). Twenty-five percent of these were less than 20/40 Snellen decimals (i.e. >0.3 LogMAR or an approximate loss of three lines of visual acuity), of which six had no light perception and an additional five could perceive light but no hand movements. In total, in 87 cases (38.8%) the definition ‘treatment failure’ (defined as <20/40 Snellen BCVA and/or the need for keratoplasty) applied.

**Table 2:** Treatment.

Type	No. (%)	Duration in days, median (range)
**Amoebicidal agents**		
*Chlorhexidine 0*.*02%*	219 (97.8)	175 (14–881)
As monotherapy	32	
Unknown	2	13
*Brolene*^*®*^ *1%*	183 (81.7)	88.5 (5–881)
As monotherapy	1	
Unknown	3	10
*PHMB 0*.*02%*	6 (2.7)	104 (32–263)
As monotherapy	2	
Unknown	3	0
**Additional medical therapy**		
*Antibiotics*	156 (69.6)	63.5 (1–819)
Unknown	3	31
*Antifungals*	126 (56.3)	43.5 (1–572)
Unknown	1	19
*Corticosteroids (after diagnosis)*	91 (40.6)	217 (5–1780)
Unknown	3	25
Duration between diagnosis and start		36 (0–708)
Unknown		6
**Maximal pain management**		data unavailable
None	90 (40.2)	
Paracetamol	23 (10.3)	
NSAIDs	41 (18.3)	
Opioids	51 (22.8)	
Unknown	19 (8.5)	
**Surgical therapy**		not applicable
*Acute intervention*		
Keratoplasty	49 (21.9)	
Enucleation	1 (0.4)	
Unknown	2 (0.9)	
*Secondary intervention*		
Keratoplasty	32 (14.3)	
Evisceration	2 (0.9)	
Unknown	1 (0.4)	
*Cataract surgery*	32 (14.3)	
Not applicable	5 (2.2)	
Unknown	26 (11.6)	

### Predictors for treatment failure

In univariable logistic regression analyses, factors influencing the results of primary AK treatment were evaluated. In Table 3, the univariable predictors with P-value <0.1 are shown. There was no univariable correlation between sex and treatment outcome, and as such a correlation was not expected clinically either, no further adjustment for sex was made in the multivariable model. Since the use of pain medication might have been a result of treatment failure rather than a predictor, this was left out of the multivariable analyses as well. The adjusted odds ratios of the four significant predictors of the multivariable model are shown in Table 4. No two-way interactions were found. All cases described were eventually referred to cornea specialists. The odds of treatment failure after direct referral to a cornea specialist by the general practitioner were 3.2 times lower than the odds for cases that were initially treated by other ophthalmologists before being referred to a cornea specialist. A higher severity stage at diagnosis, i.e. cases in which a ring infiltrate was present, increased the odds to result in treatment failure by 3.8, as compared to cases with only epitheliopathy. The odds of treatment failure after corticosteroid use before diagnosis were 3.3 times higher than the odds for cases not treated with corticosteroids. The odds of treatment failure increased with factor 1.052 per age year, resulting in a 1.66 times higher odds over ten age years.

**Table 3:** Univariable logistic regression analyses on ‘treatment failure’*.

Exposure factor	No. of patients	Odds ratio (Exp (B))	95% C.I. for Exp (B)
**Categorical**			
*Referral by*			
Other ophthalmologist (reference)	149	-	-
General practitioner	48	**0.168**	0.071–0.399
Patient	8	0.987	0.238–4.093
Other	2	0.000	0.000
*Year of presentation*			
2009 (reference)	14	-	-
2010	15	0.667	0.153–2.903
2011	31	2.444	0.664–9.000
2012	34	0.478	0.134–1.704
2013	29	0.706	0.196–2.544
2014	39	0.560	0.163–1.926
2015	47	0.469	0.139–1.578
*Severity at diagnosis*			
Stage 1 (reference)	52	-	-
Stage 2	89	0.945	0.454–1.965
Stage 3	62	**3.491**	1.608–7.581
*Pain management*			
None (reference)	85	-	-
Paracetamol	22	2.077	0.685–6.294
NSAIDs	37	**4.708**	1.961–11.301
Opioids	49	**28.385**	10.862–74.176
*Corticosteroid use before diagnosis*			
No (reference)	69	-	-
Yes	137	**4.104**	2.089–8.066
**Linear**			
Age at presentation, years	206	**1.056**	1.035–1.077
Total delay, days	185	**1.012**	1.003–1.021

*Only exposure factors with P<0,1 are shown.

Exp (B) = exponentiation of the B coefficient, C.I. = confidence interval.

**Table 4:** Multivariable logistic regression analysis on ‘treatment failure’.

Exposure factor	No. of patients	Odds ratio (Exp (B))	95% C.I. for Exp (B)
**Referral by**			
Other ophthalmologist (reference)	142	-	-
General practitioner	47	**0.311**	0.115–0.841
Patient	8	2.308	0.417–12.791
Other	1	0.000	0.000
**Severity at diagnosis**			
Stage 1 (reference)	50	-	-
Stage 2	87	1.467	0.624–3.450
Stage 3	61	**3.847**	1.544–9.584
**Corticosteroid use before diagnosis**			
No (reference)	66	-	-
Yes	132	**3.308**	1.375–7.963
**Age at presentation, years**	196	**1.052**	1.029–1.075

Exp (B) = exponentiation of the B coefficient, C.I. = confidence interval.

## Discussion

In this retrospective, multicentre case series of *Acanthamoeba* keratitis in the Netherlands, we confirm the clinical impression that the incidence of AK increased from 2009 to 2015. In this study period, an average yearly increase of 20.5% was found. Almost all cases occurred in contact lens wearers. For soft contact lens wearers, the incidence rate in 2015 was estimated to be 1 in 21,000. Taking into account that this number might be underestimated, as a result of recall bias, the incidence of AK in the Netherlands in 2015 certainly increased above the annual incidence rate of 1 in 30,000 soft contact lens wearers found by Seal [2]. One should realise that this is not a lifetime risk, but a possibly still increasing yearly risk for each eye of individual soft contact lens wearers. A similar increase in the number of AK cases was presented by Carnt et al. for the south east of the United Kingdom [4].

Most cases occurred in summer and autumn, as is shown in Fig 2. A high AK incidence in summer and autumn was also found in studies from Canada and New Zealand [10,11]. An American study reported high incidences in June and November, which correlated to the number of amoebae found in surface water [12]. These data suggest that the amoeba thrives well in high temperatures. A possible explanation is given by Lakhundi et al., who found that excystation of the *Acanthamoeba–*the transition from the dormant, double-walled cyst to the mobile trophozoite–occurs optimally in vitro at a temperature of 30°C [13].

It is widely known that contact lens wear is the main risk factor for the development of AK in developed countries. The percentage of patients wearing contact lenses in our series was 95, comparable to 93.5–96% and 96–100% in recent studies from the United Kingdom and New Zealand [11,14]. Soft contact lens wearers were slightly overrepresented in our study population: 74% versus 67% of the contact lens wearers in the general Dutch population [15]. According to several authors, good hygiene practice is less likely and at the same time more difficult for wearers of soft contact lenses, compared to rigid-gas permeable lenses, and hygiene itself is probably even more important than the type of contact lenses [4,16,17].

The multivariable regression analysis performed in this study reveals several interesting outcomes. The first is an association between a higher age at presentation and treatment failure. The risk of treatment failure increases with factor 1.052 per age year, resulting in a 1.66 times higher risk over ten age years. Besides this, patients with AK in higher stages of the disease at diagnosis were more likely to fail on treatment. Similar associations were found in earlier studies [7,14]. Carnt et al. suggested the higher prevalence of dry eyes in the elderly population to be a possible risk factor for acquiring AK, and related the higher risk of poor outcome of the disease to a possible altered host defence in elderly patients [14].

Moreover, we found a clearly elevated risk of treatment failure for patients who were initially treated with corticosteroids (odds ratio 3.31). Robaei et al. found a comparable risk (n = 163; odds ratio 3.90) and we appear to confirm the general view that corticosteroids can increase the likelihood of treatment to fail [7]. In cases misdiagnosed as HSV, corticosteroids were significantly more often prescribed, and both HSV suspicion and corticosteroid treatment before diagnosis were associated with longer doctor’s delay. HSV-misdiagnosis occurred in 58%, similar to the proportion of misdiagnosed cases in the population of Robaei et al [7]. This demonstrates the difficulty in differentiating between HSV and AK, and the importance of clinical inspection by an experienced ophthalmologist.

Carnt et al. suggest that corticosteroids may be used after diagnosis in cases with a persistent inflammatory reaction, provided that the keratitis has been treated by amoebicidal therapy for at least two weeks and this therapy is continued while using steroids [14]. As surgical intervention was one of the criteria for treatment failure in our study, and corticosteroids were always prescribed after penetrating keratoplasty, a correlation between failure and corticosteroid treatment after diagnosis is obvious and confounded. It has therefore been left out of the multivariable regression analysis. It has been hypothesised that steroids stimulate excystation of the amoeba by optimising its environment, and it would be interesting to investigate whether anti-*Acanthamoeba* treatment is more effective after a short course of steroids [18].

An interesting finding was the negative correlation between direct general practitioner referral to a cornea subspecialist and treatment failure, suggesting a protective value. Since peripheral ophthalmologists have less experience treating AK than cornea specialists, recognising the disease and differentiating it from HSV keratitis can be challenging. The finding that general practitioners prescribed corticosteroids less often than peripheral ophthalmologists probably contributes to this protective effect.

The retrospective design of this study implies the possibility of missed cases, either because they were never recognised as AK, or because the approached ophthalmologists did not register all cases. It is unexpected, however, that AK cases resolved without reaching one of the cornea specialists in our country. The Netherlands is a small and densely populated country with a relatively high (sub)specialist availability. It is therefore assumed that the number of missed cases is small. Additionally, the retrospective nature of the study certainly leads to missing data.

As compared to other studies the criteria for treatment failure might be considered strict [7,14]. The cut-off point of a BCVA of <20/40 Snellen decimals had been chosen because this is a threshold for surgical intervention in many ophthalmological diseases and it is the required minimum visual acuity for the driving license in Europe [19].

In conclusion, like in the UK, the incidence of AK in the Netherlands is undoubtedly rising. This is a worrisome evolution, as treatment failure is not uncommon. By increasing the awareness of the disease amongst contact lens wearers and ophthalmologists, delay until diagnosis and corticosteroid use before diagnosis can hopefully be diminished. Further studies to prevent AK may contribute to eventually turn down the rising incidence.

## Supporting information

S1 File Database containing relevant raw data. (XLSX)
